# Ciprofloxacin binding to GyrA causes global changes in the proteome of *Pseudomonas aeruginosa*

**DOI:** 10.1093/femsle/fny134

**Published:** 2018-05-28

**Authors:** Hannah Jedrey, Kathryn S Lilley, Martin Welch

**Affiliations:** Department of Biochemistry, Tennis Court Road, Cambridge, CB2 1QW, UK

**Keywords:** ciprofloxacin, antibiotic resistance, *Pseudomonas aeruginosa*, Proteomic, DNA Gyrase, GyrA

## Abstract

Ciprofloxacin is one of the most widely-used antibiotics, and has proven especially effective at controlling infections associated with the opportunistic human pathogen, *Pseudomonas aeruginosa*. In this work, we show that sub-inhibitory concentrations of ciprofloxacin induce discrete changes in the intracellular proteome. Central metabolism and cell envelope-associated functions are particularly affected. In spite of the low magnitude of the intracellular proteomic changes, we found that sub-lethal concentrations of ciprofloxacin had substantial effects on motility and exoprotein secretion. Crucially, the proteomic and phenotypic modulations that we observed were absolutely dependent upon the presence of wild-type GyrA; an isogenic strain of *P. aeruginosa* carrying a ciprofloxacin-insensitive form of GyrA (a T83→I mutant) did not display ciprofloxacin-dependent changes unless complemented with wild-type *gyrA in trans*. These results show that the diverse effects of sub-inhibitory ciprofloxacin on the cell are routed through its primary target in the cell, DNA gyrase.

## Teaser

Binding of the “blockbuster” antibiotic, ciprofloxacin, to one of the DNA gyrase subunits in a critical priority pathogen (Pseudomonas aeruginosa) leads to global changes affecting many aspects of bacterial physiology.

## INTRODUCTION

*Pseudomonas aeruginosa* is an opportunistic pathogen responsible for causing a variety of human disease states, especially among immunocompromised individuals. Indeed, in 2017, the World Health Organisation (WHO) named *P. aeruginosa* as a critical priority pathogen against which new antimicrobial interventions are urgently required. One of the most effective antibiotics used to treat *P. aeruginosa* infections is the second generation fluoroquinolone, ciprofloxacin (CIP) (Wise, Andrews and Edwards 1983). Introduced in 1987, CIP proved so effective at treating a wide range of bacterial infections that it rapidly joined the WHO list of medicines essential for basic healthcare. CIP has a broad spectrum of action with good tissue penetration, oral absorption and favourable phamacokinetics, making it ideal for the treatment of a wide range of infections. Crucially, the presence of the cyclopropane moiety on the N atom of the heterocycle in CIP increases its activity (compared with first generation fluoroquinolones such as norfloxacin) against *P. aeruginosa* by a factor of four.

In Gram-negative bacteria, the primary target of CIP is DNA gyrase (a tetramer comprised of 2 GyrA and 2 GyrB molecules), although topoisomerase-IV (ParC) is a secondary target and can be inhibited by higher concentrations of the drug. Quinolones do not bind to the gyrase enzyme alone, but rather, to the gyrase-DNA complex (Hooper 2001). The region of GyrA spanning residues 51–106 is known as the quinolone-resistance determining region (QRDR), since mutations within this sequence decrease the susceptibility of the organism to fluoroquinolone inhibition (Yoshida *et al.*^1990^). Not surprisingly, this stretch of sequence maps to the recognition helix responsible for binding DNA. Among QRDR mutants, substitutions at residue 83 are frequently associated with CIP resistance in both P. aeruginosa and Escherichia coli clinical isolates (reviewed by Hooper 1999). In many CIP-resistant *gyrA* mutants, residue 83 is replaced by a bulkier, non-polar residue that is thought to impede DNA-binding to gyrase, and hence, also fluoroquinolone binding. Interestingly in E. coli, residue 83 is a serine, whereas in *P. aeruginosa* it is the bulkier threonine; this may correlate with the higher intrinsic resistance of *P. aeruginosa* to CIP compared with E. coli (Fabrega *et al.*^2009^).

Previous transcriptomic analyses have revealed that exposure of *P. aeruginosa* cultures to sub-minimal inhibitory concentrations of CIP (sub-MIC_CIP_) brings about large-scale changes in gene expression, with hundreds of transcripts showing altered abundance in CIP-treated cultures (Brazas and Hancock 2005). This raises the question of whether these modulations are a direct consequence of GyrA inhibition, or whether CIP also has ‘off target’ effects. To address this, in the current work, we used quantitative 2D-difference gel electrophoresis (2D-DiGE) to compare the adaptive response of wild-type *P. aeruginosa* and an isogenic T83→I *gyrA* mutant to sub-MIC_CIP_. Our results suggest that sub-MIC_CIP_ has significant impacts on central metabolism and protein secretion, and these effects are largely dependent upon the presence of a CIP-sensitive *gyrA* allele. Very few off-target effects are apparent, even at very high concentrations of CIP.

## MATERIALS AND METHODS

### Bacterial strains used

The PAO1 employed in the current study was a generous gift from Barbara Iglewski (University of Rochester, NY). HGS4 is a derivative of that PAO1 strain.

### Cloning of *gyrA* for complementation

A 3 kb DNA fragment including the *gyrA* coding region and its associated Shine-Dalgarno sequence was PCR-amplified (LT Polymerase, primers GyrApF2 (5’-TGAGAGCTCCCACCAGAAAAAGGAACCAG-3’) and GyrApR (5’-AGTCAACCCGGGAGAAATTGAAGGCTCGCTTG-3’)). The fragment was sequentially digested with *Sma*I/*Sac*I and ligated with pUCP20 (West *et al.*^1994^) that had been digested with the same enzymes. This yielded pGyrA.

### Media

Unless otherwise stated, all cultures were grown in AGSY medium (Stickland *et al.*^2010^).

### Minimum inhibitory concentration determination

Minimum inhibitory concentrations (MICs) were determined using E-Test strips laid onto a lawn of the relevant strain grown on the surface of AGSY-agar plates.

### Whole cell 2D-DiGE Proteomic Analysis

2D-DiGE Biological Variance Analysis (BVA; GE Healthcare) was used to analyse the proteomic changes accompanying treatment of wild-type *P. aeruginosa* and the isogenic T83→I *gyrA* mutant to sub-MIC_CIP_. Four independent biological replicates of wild-type PAO1 (each treated with 0, 0.05, 0.075 μg/ml CIP) and HGS4 (0, 0.075, 0.25 μg/ml CIP) cultures were analysed. When present, sub-MIC_CIP_ was added at the start of each growth curve. The cultures were grown in AGSY medium with good aeration, as previously described (Stickland *et al.*^2010^) and harvested for proteomic analysis when they reached early stationary phase (OD_600_ = 6, ca. 5–6 hrs after inoculation). The 2D-DiGE, spot identification and statistical analyses were carried out as previously described (Stickland *et al.*^2010^).

### Secretome protein extraction

Protein from the secretome of PAO1 (pUCP20), HGS4 (pUCP20) and HGS4 (pGyrA) cultures grown with or without 0.075 μg/ml CIP was analysed. Extracellular material was collected at OD_600_ = 6 from cultures grown in AGSY supplemented with 50 μg/ml carbenicillin (to maintain the introduced pUCP20 or pUCP20-derived plasmids). Secreted proteins were precipitated from filter-sterilised culture supernatant by addition of 12.5% (w/v, final concentration) trichloroacetic acid (TCA), essentially as described previously (Swatton *et al.*^2016^). The mixture was left on ice overnight and precipitated proteins were collected by centrifugation (12,000 × *g*, 4°C). The pellet was washed three times in 80% acetone, air-dried, and then resuspended in 50mM Tris-HCl (pH 6.8) containing 10% glycerol and 2% SDS. The pH was adjusted to ∼ pH 7 and the protein concentration was determined (BioRad detergent-compatible quantification kit). Proteins were resolved by SDS-PAGE (10–20% gradient gels).

### Swarming

For swarming motility assays, 5 μl of an overnight culture diluted to an OD of 0.1 was carefully pipetted onto the surface of 0.7% (w/v) Eiken agar prepared in AGSY medium and incubated overnight. The plates were incubated at 30°C so that motility zones were not too large to measure. Swarming motility was qualitatively assessed by how far the cells had travelled from the point of inoculation and for tendril formation.

## RESULTS

### Isolation of a spontaneous ciprofloxacin-resistant *gyrA* mutant

**Figure 1.**
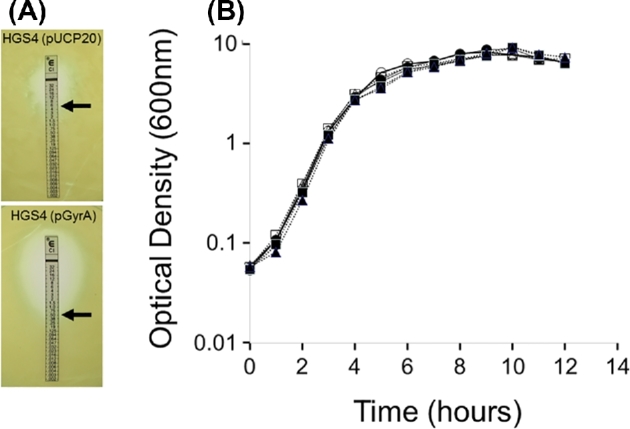
(A) E-Test strips showing the MIC_CIP_ of HGS4 (the *gyrA* T83→I mutant) containing either pUCP20 (upper panel) or pGyrA (lower panel). (B) Growth of PAO1 in the presence of increasing concentrations of CIP. Open Symbols/solid line; wild-type, filled symbols/dashed line; HGS4. (○); untreated PAO1, (Δ) 0.05 μg/mL CIP (PAO1), (■); 0.075 μg/mL CIP (PAO1), (•); untreated HGS4, (▪); 0.075 μg/mL CIP (HGS4), (▴); 0.25 μg/mL CIP (HGS4).

Wild-type PAO1 (MIC_CIP_ = 0.25 μg/ml in AGSY medium) was inoculated into AGSY medium containing 1 μg/ml CIP and incubated with good aeration at 37°C. After 24 hours, the culture had become cloudy, indicating the emergence of spontaneous CIP-resistant mutant(s). Samples from the 24 hours culture were serially-diluted and spread onto agar plates containing 1 μg/ml CIP to yield single colonies. A range of colony morphotypes were observed. Targeted PCR/sequence analysis revealed that some of these colonies contained *nfxB* mutations (which lead to up-regulation of the normally cryptic *mexCD*-*oprJ* CIP efflux pump (Poole *et al.*^1996^; Jeannot *et al.*^2008^)), whereas others were wild-type for *nfxB*. These results indicate that even within a single overnight culture, CIP-resistance can arise due to different mutations. A single CIP-resistant colony that did not contain an *nfxB* mutation was picked for further analysis. Targeted PCR amplification and sequencing of the QRDR of *gyrA* revealed that the amplicon contained a C→T transition converting the Thr83 codon (ACC) in the *gyrA* ORF to an Ile codon (ATC). The T83→I alteration is a well-established cause of CIP-resistance (Hooper 1999; Hooper 2001). This *gyrA* mutant was named HGS4. The QRDR of the *parC* locus was also amplified and sequenced to confirm that no *parC* mutations were present in HGS4. To further demonstrate that the increased CIP-resistance of HGS4 was solely due to the *gyrA* mutation, the *gyrA* ORF and its associated Shine-Dalgarno sequence was PCR-amplified from wild-type genomic DNA and cloned into pUCP20, generating pGyrA. The MIC_CIP_ of pGyrA-complemented HGS4 was restored to near wild-type levels (ca. 0.5 μg/mL), whereas the MIC_CIP_ of HGS4 containing pUCP20 remained high (MIC_CIP_ = 4–6 μg/ml (Fig. 1A).

### Proteomic adaptations of *P. aeruginosa* to ciprofloxacin

CIP concentrations of ≤ 0.075 μg/ml (corresponding to 0.3 × MIC_wild-type_) did not affect the growth of PAO1 in AGSY, although concentrations greater than this led to a reduction in growth rate and stationary phase optical density. In contrast, the *gyrA* mutant (HGS4) tolerated concentrations of 0.25 μg/ml CIP (1 × the wild-type MIC_CIP_) without any apparent effect(s) on growth (Fig. 1B). For proteomic analysis, we therefore challenged wild-type PAO1 cultures with 0.05 μg/ml CIP and 0.075 μg/ml CIP (0.2 × MIC and 0.3 × MIC, respectively), whereas the HGS4 cultures were challenged with 0.075 μg/ml CIP and 0.25 μg/ml CIP. These concentrations were selected for study (i) to allow comparison of the proteomic changes brought about at the highest non-growth rate impairing concentration of CIP in the wild-type (0.075 μg/ml) with the target site mutant (HGS4), and, (ii) to examine whether a lethal concentration (for the wild-type strain) of CIP elicits any proteomic changes in the target site mutant (0.25 μg/ml CIP is equal to the MIC of the wild-type strain). Untreated cultures served as controls. Whole cell lysates were analyzed by 2D-difference gel electrophoresis (2D-DiGE) using a BVA protocol, allowing smaller fold-changes to be reliably determined (Stickland *et al.*^2010^). This work was carried out as part of a larger BVA, which also included comparison of the wild-type with an *nfxB* mutant. Mutations in *nfxB* confer resistance to CIP by up-regulating expression of the MexCD-OprJ efflux pump (Shiba *et al.*^1995^; Poole *et al.*^1996^). Our findings assessing the impact of *nfxB* mutation on the proteome have been previously published (Stickland *et al.*^2010^) and the master gel for the BVA was presented in that study. Until now, the outcome of the second half of that BVA study (examining the impact of CIP on the wild-type proteome) has not been reported.

**Figure 2.**
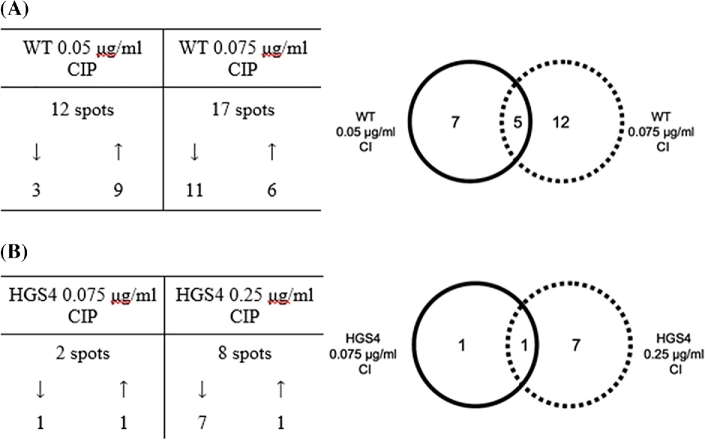
Numbers of protein spots modulated (*P* ≤ 0.01) in the cell-associated proteome of (A) the wild-type or (B) HGS4 following exposure to the indicated concentration of CIP (all modulations relative to the untreated wild-type or HGS4 proteome, respectively). The tables show whether the spots were up- or downregulated. For example, of the 12 spots that were modulated by 0.05 μg/mL CIP in the wild-type, 3 showed decreased abundance in the treated samples (cf. the untreated samples) and 9 showed increased abundance in the treated samples.

The average ratio of standardized protein spot abundance was calculated for each of the spots in the filtered dataset. To our surprise, even the highest concentration of CIP tested had little effect on the global proteome; only 24 protein spots (out of a total of 853 spots) were significantly (*P* ≤ 0.01) modulated in the CIP-treated wild-type samples compared with the untreated controls (Fig. 2, upper panel). Interestingly, not all of the proteins that were modulated at the lower concentration of CIP (0.05 μg/ml) were also modulated at the higher concentration (0.075 μg/ml) of CIP (although there was some overlap). This may indicate that the cell implements a ‘graded’ response to the antibiotic, possibly reflecting the impact of differential gyrase inhibition on gene expression profiles (DNA gyrase-dependent supercoiling is known to influence transcription (reviewed by Dorman and Dorman 2016)) or differential stimulation of reactive oxygen species production (see Discussion). The proteome of untreated HGS4 was essentially indistinguishable from that of the untreated wild-type (*data not shown*). This indicates that the T83→I substitution in HGS4 GyrA does not in itself lead to obvious cellular perturbations. Also, fewer and smaller changes were elicited by addition of CIP to HGS4 cultures (Fig. 2, lower panel). None of the spots modulated by CIP in HGS4 overlapped with the CIP-modulated spots in the wild-type. These data suggest that in the wild-type, most of the observed changes are dependent upon the interaction of CIP with GyrA.

### Multivariate analysis of the proteomic data

**Figure 3.**
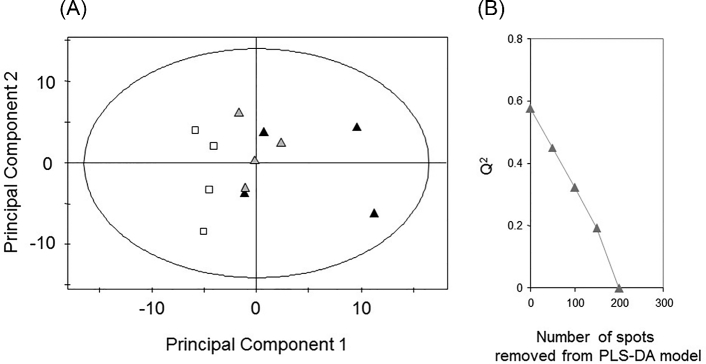
Sub-MIC_CIP_ causes global changes in the proteome. (A) PCA scores plot showing samples of the wild-type treated with no CIP (open squares), 0.05 μg/ml CIP (gray triangles), and 0.075 μg/ml CIP (black triangles). Each data point represents the eigenvalues (PC1 and PC2, respectively) of the corresponding normalized eigenvectors. (B) A PLS-DA model was constructed in which the first component accounted for 83% of the proteomic differences between untreated and treated (0.075 μg/ml CIP) samples. The model had good predictive power (Q2 = 58%). In an iterative process involving removal of the top 50 spots, followed by recalculation of the model, we found that the model collapsed (i.e. lost predictive power) after removal of 200 ± 25 spots. This indicates that sub-MIC_CIP_ treatment of wild-type *P. aeruginosa* alters the abundance of two hundred protein spots.

Our finding, that only small numbers of protein spots were significantly modulated by sub-MIC_CIP_ exposure, contrasts strongly with earlier microarray experiments which reported that hundreds of transcripts are modulated by sub-MIC_CIP_ (Brazas and Hancock 2005). One possible explanation for this disparity is that many of the proteomic changes elicited by CIP treatment are subtle and escape detection based on univariate statistical analyses, which only assess pairwise comparisons. We therefore re-analysed the proteomic dataset using an unsupervised multivariate approach, principal components analysis (PCA). A scores plot of the first two principal components in the model (Fig. 3A) shows that the CIP-treated wild-type samples show a trend towards segregating away from the untreated samples, with the higher CIP concentration driving greater separation. To further tease out the drivers underpinning these separations, a more sensitive, supervised approach (partial least squares-discriminant analysis, PLS-DA) was employed. The first component accounted for the majority (83%) of the differences between the sample types (*data not shown*). To establish how many protein spots contributed towards this separation, we iteratively removed spots from the PLS-DA model until the robustness (Q^2^ value) of the model collapsed (Fig. 3B) (Karp, Griffin and Lilley 2005). This happened after ∼200 ± 25 protein spots were removed, indicating that around 23% (200/853 spots) of the observed proteome was modulated by sub-MIC_CIP_ treatment. Our data are therefore broadly consistent with earlier microarray-based approaches indicating that CIP-induced alterations involve hundreds of gene products. Many of these modulations were subtle and involved low-fold changes, consistent with maintenance of a relatively constant intracellular proteomic steady state.

### Identities of the modulated spots

A selection of the CIP-modulated spots identified in both the multivariate and *t*-test analyses were excised from the gels and tryptically digested to yield peptide fragments. These peptides were then identified by LC-MS/MS analysis and MASCOT searching. Spots which yielded identities for two or more proteins (i.e. ‘mixture hits’ arising from overlapping spots) were excluded from further analysis. The identified proteins in the CIP-modulated spots from the wild-type and HGS4 samples are shown in Table 1 and Table S1, respectively. There was no overlap between the identified proteins in the wild-type and HGS4 samples. The modulated spots were classified into functional groups according to their PseudoCAP ontology (www.pseudomonas.com/). Notably, CIP treatment resulted in the modulation of several enzymes catalyzing reactions that are either part of the TCA cycle or that feed in/out of the TCA cycle. These include pyruvate kinase (PykA), the superoxide-insensitive isozyme of fumarate hydratase (FumC1), two differentially modulated isozymes of *iso*citrate dehydrogenase (PA2623 (*icd*, down-regulated) and PA2624 (*idh*, up-regulated)) and malic enzyme. Consistent with the up-regulation of FumC1 (indicating oxidative stress), we also observed that the house-keeping catalase (KatA) was up-regulated. Other notable modulated proteins included the chaperonin, GroEL (multiple spots up-regulated), the flagellar capping protein, FliD, proteins involved in cell envelope maintenance such as MurE (UDP-MurNac-tripeptide synthetase; up-regulated) and MreB (a DnaK-like molecular chaperone involved in determining cell shape; down-regulated) and a DegP/HtrA-like serine protease (MucD) involved in the control of alginate production and response of envelope stress (down-regulated).

**TABLE 1.** Identity of selected spots whose abundance was modulated in wild-type PAO1 cultures by sub-MIC_CIP_.

			Fold change		Identified in:		
PA number	Gene	Modulation	0.05 μg/ml CIP	0.075 μg/ml CIP	*t-*test	PLS-DA	Protein
Amino acid biosynthesis and metabolism, translation, post translational modification and degradation							
5013	*ilvE*	↑	1.67		○	•	Branched-chain amino acid transferase
2709	*cysK*	↓				•	Cysteine synthase A
0400	*(metB)*	↑				•	Probable cystathionine gamma-lyase
1750		↓		–1.21	○		Phospho-2-dehydro-3-deoxyheptonate aldolase
5038	*aroB*	↓		–1.37	○		3-dehydroquinate synthase
0956	*proS*	↑	1.67		○		Prolyl-tRNA synthetase
Central intermediary metabolism, energy metabolism and carbon compound metabolism							
2623	*icd*	↓	–1.3		○		*Iso*citrate dehydrogenase
2624	*idh*	↑	1.78		○	•	*Iso*citrate dehydrogenase
4329	*pykA*	↑		1.47	○		Pyruvate kinase II
4470	*fumC1*	↑				•${^{*}}$	Fumarate hydratase (fumarase)
5046		↓		–1.33	○		Malic enzyme
5554	*atpD*	↑				•	ATP synthase beta chain
Putative enzymes							
4079		↓		–1.78	○	•	Probable dehydrogenase
Adaption and protection, chaperones and heat shock							
4236	*katA*	↑		1.48	○		Catalase
4385	*groEL*	↑				•${^{*}}$	GroEL chaperonin
Cell wall/LPS/capsule—includes other categories: cell division, motility and attachment and secreted factors							
4417	*murE*	↑	1.32	1.78	○	•	UDP-MurNac-tripeptide synthetase
4481	*mreB*	↓				•	Rod shape-determining protein MreB
1094	*fliD*	↓				•	Flagellar capping protein FliD
0766	*mucD*	↓				•	Serine protease MucD precursor
Transport of small molecules							
4595	*(yjjK)*	↓		–1.41	○		Probable ATP-binding component of ABC transporter
1810	*nppA2*	↑				•	Peptidyl nucleoside antibiotic transporter (periplasmic binding protein)
Nucleotide biosynthesis and metabolism							
1013	*purC*	↓		–1.43	○		Phosphoribosylaminoimidazole- succinocarboxamide synthase

A protein modulation was designated as significant if identified by *t*-test (*P* ≤ 0.01). *T*-tests were carried out comparing untreated wild-type samples with wild-type samples that had been treated with the indicated concentration of CIP. The magnitude and direction of modulation is shown as fold-change (for proteins identified by *t*-test) and by the arrows. A protein identification was considered significant if the MASCOT score was > 106 (the equivalent of two different significant peptide ion matches). * Indicates that a protein was identified in more than one spot.

Of the identified proteins modulated in HGS4, most were down-regulated, and most of these were affected only at the higher concentration of CIP tested (0.25 μg/ml), perhaps indicative of off-target or secondary target effects of CIP. Most notably, GyrB was down-regulated, as was the AAA + ATPase family chaperone, ClpB.

### Phenotypic consequences of CIP-challenge

**Figure 4.**
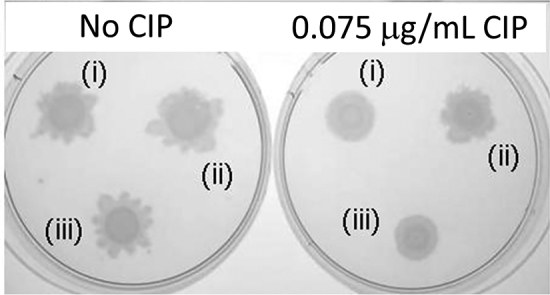
Ciprofloxacin depresses tendril formation during surface swarming by inhibiting GyrA. Aliquots of culture containing the indicated strains were spotted onto 0.7% (w/v) Eiken agar plates and left at 30°C to allow swarm development. (i) WT (pUCP20), (ii) HGS4 (pUCP20) and (iii) HGS4 (pGyrA). Note that CIP depresses tendril formation in the wild-type but not in the *gyrA* T83→I mutant, HGS4. However, tendril formation is depressed in HGS4 following expression of wild-type *gyrA* in trans.

To investigate the possible consequences of the proteomic modulations, we examined a selection of phenotypes that might be affected by these. Motility plays a key role in infection, and our proteomic analysis showed that the flagellar capping protein, FliD, was down-regulated in the presence of CIP. Consistent with this, we noted that the surface swarming ability of wild-type *P. aeruginosa* was impaired in the presence of 0.075 μg/ml CIP (Fig. 4). No impairment of motility was seen in the CIP-treated HGS4 mutant. However, when HGS4 was complemented with pGyrA, surface swarming once again became sensitive to the presence of CIP. We conclude that the effect of CIP on motility is dependent on the presence of a susceptible *gyrA* allele in the wild-type.

**Figure 5.**
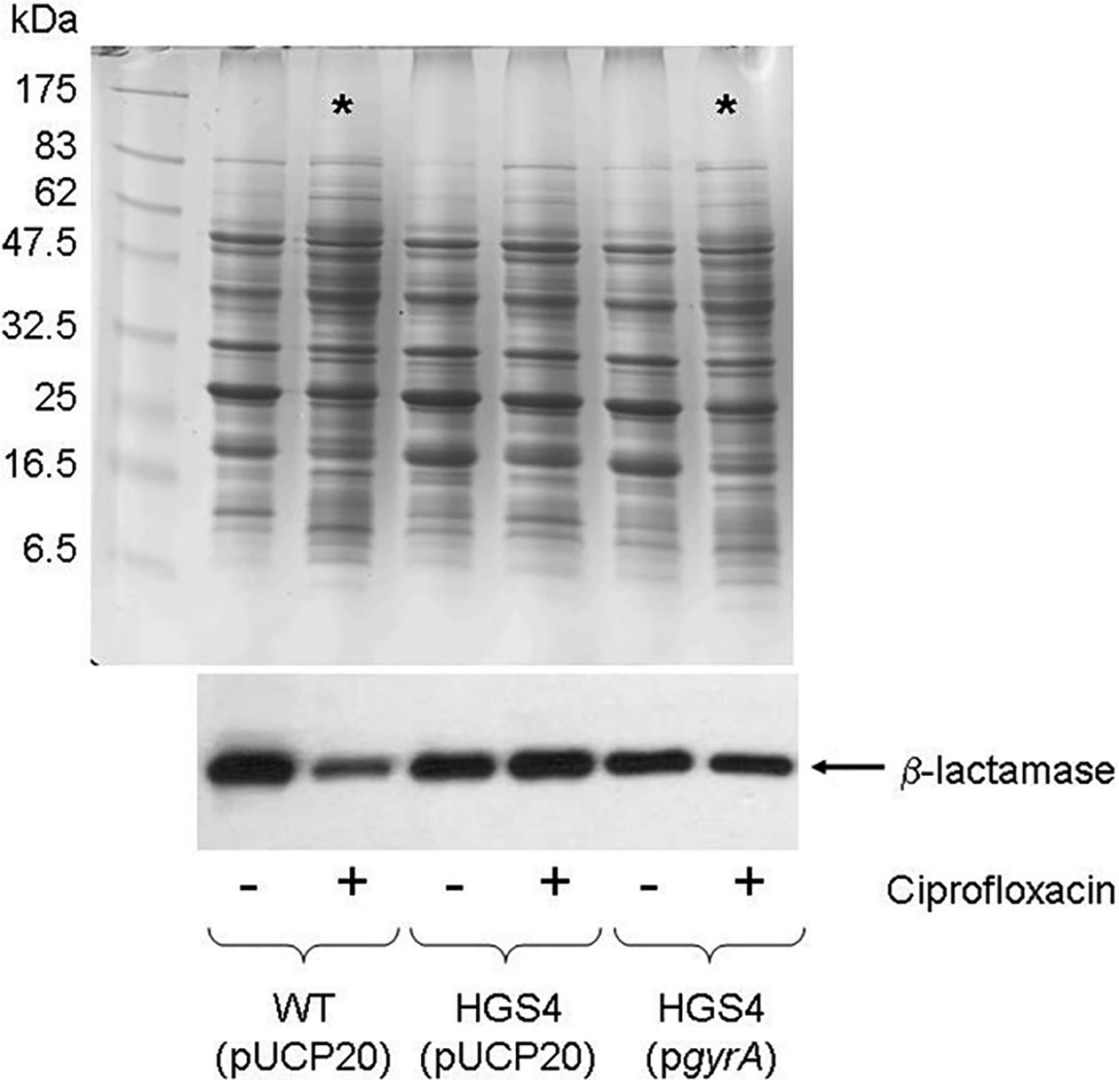
Sub-MIC_CIP_ causes changes in the *P. aeruginosa* secretome. The figure shows a 1D SDS-PAGE gel stained with Coomassie Brilliant Blue (upper panel) of secretome samples from cultures of the indicated strains grown ± 0.075 μg/ml CIP. Below this (lower panel) is a Western blot of the same samples probed with an antibody (from 5prime3prime) reactive against the β-lactamase encoded on the pUCP20 plasmid. Asterisks indicate lanes where the secretome is clearly very different compared with the untreated wild-type control.

The altered motility elicited by CIP, as well as the identification of several CIP-modulated proteins associated with cell envelope physiology made us wonder whether protein secretion might be affected by the drug. Indeed, earlier workers have shown that CIP treatment of *P. aeruginosa* biofilms alters the spectrum of proteases secreted (Ołdak and Trafny 2005). Therefore, we used 1D SDS-PAGE to analyse the secretome of wild-type PAO1 (pUCP20), HGS4 (pUCP20) and HGS4 (pGyrA). Sub-MIC CIP caused clear alterations in the profile of proteins secreted by cultures of wild-type PAO1, but no obvious major alterations in the protein profile secreted by HGS4 (Fig. 5, upper panel). However, when HGS4 was complemented with pGyrA, the secretome once again became sensitive to CIP addition. These data suggest that sub-MIC_CIP_ treatment impacts upon protein secretion by *P. aeruginosa* through its effects on GyrA. To confirm that the observed secretome alterations were not due to increased CIP-dependent cell lysis, we used Western blotting to assess β-lactamase levels in the secretome samples. β-lactamase is constitutively expressed from the pUCP20 plasmids in each strain and is a periplasmic enzyme. Therefore, release of β-lactamase is a good indicator of cell envelope integrity. There was no evidence of enhanced cell lysis in the CIP-treated samples (Fig. 5, lower panel).

## DISCUSSION

In this study, we show that the adaptive response to sub-MIC_CIP_ involves a small number of moderate (<2-fold) changes in intracellular protein abundance, and a much larger number of lower-fold modulations. These subtle modulations are consistent with maintenance of a relatively constant overall intracellular steady state proteome. Larger CIP-induced proteomic modulations were seen in the exoproteome, which is presumably subject to less stringent control of its composition. However, we also show that in spite of the low magnitude of the intracellular proteomic modulations, some of these have clear phenotypic consequences. We further show that these sub-MIC_CIP_-induced phenotypic and proteomic changes are absolutely dependent upon the drug interacting with wild-type (CIP-sensitive) GyrA. Indeed, even at a concentration of CIP that completely prevents the growth of wild-type cultures (i.e. 0.25 μg/ml, equal to the MIC_CIP_ of PAO1), the *gyrA^T83I^* mutant showed very few proteomic changes.

In a previous study, Brazas *et al* used microarray analysis to investigate the effects of sub-MIC_CIP_ on the global gene expression profile of *P. aeruginosa* (Brazas and Hancock 2005). Those workers found that the transcripts from 941 genes were affected in the presence of 0.3 × MIC_CIP_. In contrast, we observed that only around 200 protein spots were affected by 0.3 × MIC_CIP_. However, this may simply reflect differences in the relative sampling efficiency (i.e. gene products detectable) of proteomics compared with transcriptomics. Moreover, 60% of the gene products reported in the current study were not reported as being modulated by Brazas *et al.* and of the gene products that were common to both studies, only four (GroEL, Icd, IlvE and PA4595) were modulated in the same direction. It seems likely that the main driver for the disparity between the two studies lies in biological differences, probably related to the different growth conditions employed (Brazas *et al* employed oligopeptide-based LB for their study, while we used alanine-glycerol-salts medium).

Why should inhibition of DNA gyrase lead to pleiotropic changes in the cell? Davies and colleagues have shown that the cellular response to many antibiotics exhibits the property of hormesis, suggesting that these compounds may have evolved as intercellular signals (Davies, Spiegelman and Yim 2006). A variation on this scenario is that low concentrations of antibiotic may induce protective measures that ‘prime’ the cell ready to respond higher concentrations of the same compound. While this may indeed be the case, we note that CIP is an entirely synthetic antibiotic, so there is no reason to believe that the cell will have evolved mechanisms to respond specifically to its presence. However, just as the ribosome can titrate translational inhibitors, we suspect that DNA gyrase may monitor the general ‘state of health’ of the genome and allow the cell to adapt accordingly. Two possible generic gyrase-dependent adaptatory mechanisms present themselves. First, the observed proteomic modulations may arise as a consequence of altered gene expression brought about directly by GyrA inhibition. Indeed, DNA gyrase-dependent supercoiling is known to affect the expression of many genes (Dorman and Dorman 2016). In this scenario, adaptations which protect against the effects of GyrA inhibition would be hardwired into the global gyrase regulon. The second mechanism is indirect and involves adaptive measures that protect against the bactericidal effect(s) of the antibiotic. Kohanski *et al.* (2007) have proposed that all bactericidal antibiotics (including CIP) exhibit a common mechanism of killing. In this model, inhibition of the target molecule activity leads, *via* transient stimulation of superoxide production, to a runaway Fenton reaction and subsequent accumulation of lethal hydroxyl radicals. In this case, the proteomic changes induced by exposure to low concentrations of the antibiotic would be expected to comprise part of the cell's adaptive response to the imposed (antibiotic-dependent) oxidative stress. While we cannot distinguish between these two possible modes of action, it is clear from Table 1 that enzymes involved in the response to oxidative stress (KatA, FumC1) are indeed up-regulated by exposure to sub-MIC_CIP_.

Another set of potentially related gene products that were modulated by sub-MIC_CIP_ are involved in metabolism. Malic enzyme (PA5046), which catalyses the oxidative decarboxylation of malate to yield pyruvate, was down-regulated, whereas pyruvate kinase (PykA) was up-regulated. This suggests that a net effect of CIP exposure is to decrease the flow of metabolites out of the TCA cycle (e.g. for gluconeogenesis) and increase the flux of carbon going into the cycle. In addition, the *iso*citrate dehydrogenase encoded by *icd* was down-regulated whereas the NADP-dependent *iso*citrate dehydrogenase isozyme encoded by *idh* was up-regulated. Little is known about these two isozymes in *P. aeruginosa*, although it seems likely that they are differentially regulated. Crucially, the products of the *iso*citrate dehydrogenase reaction are α-*keto*glutarate and NADPH, both of which play an important role in the response to oxidative stress (Mailloux *et al.*^2007^; Spaans *et al.*^2015^). Taken together, it seems likely that exposure to sub-MIC_CIP_ leads to an accumulation of reactive oxygen species (as per the Kohanski model), and that in response to this, the cell increases flux around the TCA cycle to generate α-*keto*glutarate and NADPH. Consistent with this, we further note that the cystathionine γ-lyase, MetB, was also up-regulated upon exposure to CIP. Cystathionine γ-lyase breaks down cystathionine to yield α-*keto*glutarate.

Interestingly, one of the gene products (PA4595) identified in both this and a previous transcriptomic study (Brazas and Hancock 2005) as being down-regulated in the presence of sub-MIC_CIP_ is a predicted ATP-binding component of an ABC transporter. The PA4595 gene is located immediately adjacent to a cell envelope stress-inducible *nfxB*-like repressor, *esrC*. *EsrC* and *nfxB* flank the *mexCD*-*oprJ* multidrug efflux pump, well-known for being a major determinant of CIP resistance (Stickland *et al.*^2010^), and both regulators are known to affect expression of the pump under different conditions (Purssell *et al.*^2015^). The functional relationship, if any, between PA4595 and EsrC/MexCD-OprJ/NfxB is not clear, and the location of PA4595 adjacent to this known CIP resistance determinant may be entirely coincidental. However, we previously showed that the protein encoded by PA4595 is up-regulated in an *nfxB* mutant (Stickland *et al.*^2010^), suggesting a possible functional linkage.

In summary, we have shown that exposure to sub-MIC_CIP_ leads to re-modelling of the intracellular proteome. This re-modeling has phenotypic consequences, and is dependent on the binding of CIP to a sensitive GyrA protein. Many of the proteomic changes that are elicited by sub-MIC_CIP_ appear to be linked with the cellular response to oxidative stress, although we cannot exclude the possibility that some of these modulations reflect an altered DNA supercoiling state in the cell as the DNA gyrase becomes progressively more inhibited by higher concentrations of CIP.

## Supplementary Material

Supplementary Data
