# Supplementary material for: Ciprofloxacin binding to GyrA causes global changes in the proteome of Pseudomonas aeruginosa
Source: FEMS Microbiol Lett. 2018 May 28;365(13):fny134. doi: 10.1093/femsle/fny134 (PMC5995189; doi:10.1093/femsle/fny134)
Supplement: Supplementary Data [file fny134_supplemental_files.docx]

**TABLE S1.** Identity of selected spots whose abundance was modulated in HGS4 cultures by sub-MIC_CIP_.

| **PA number** | **Gene** | **Modulation** | | **Fold Change** | | **Identified in *t*-test** | **Protein identification** |
| --- | --- | --- | --- | --- | --- | --- | --- |
|  |  |  |  | 0.075 μg/ml CIP | 0.25 μg/ml CIP |  |  |
| **Amino acid biosynthesis and metabolism** | | | | |  |  |  |
| 4180 |  | ↓ |  | | -1.24 | ○ | Probable acetolactate synthase (large subunit) |
| **Translation, post translational modification and degradation** | | | | | | |  |
| 3083 | *pepN* | ↓ | |  | -1.17 | ○ | Aminopeptidase N |
| 4542 | *clpB* | ↓ | | -1.27 | -1.27 | ○ | ClpB chaperone |
| **Putative enzymes** | | | | | |  |  |
| 0508 |  | ↓ | |  | -1.15 | ○ | Probable acyl-CoA dehydrogenase |
| **Transport of small molecules** | | | |  |  |  |  |
| 0888 | *aotJ* | ↑ | |  | 1.17 | ○ | Arginine /ornithine binding protein |
| **DNA replication and repair** | | | |  |  |  |  |
| 0004 | *gyrB* | ↓ | |  | -1.25 | ○ | Gyrase subunit B |

**Table S1.** A protein modulation was designated as significant if identified by *t*-test (*p* ≤ 0.01). *T*-tests were carried out comparing untreated HGS4 samples with HGS4 samples that had been treated with the indicated concentration of CIP. The magnitude and direction of modulation is shown as fold-change (for proteins identified by *t*-test) and by the arrows. A protein identification was considered significant if the MASCOT score was >106 (the equivalent of two different significant peptide ion matches). ***** Indicates that a protein was identified in more than one spot.
